# Adaptation and interaction of saxicolous crustose lichens with metals

**DOI:** 10.1186/1999-3110-55-23

**Published:** 2014-02-04

**Authors:** Ole William Purvis

**Affiliations:** Camborne School of Mines, College of Engineering, Mathematics and Physical Sciences, University of Exeter, Cornwall Campus, Penryn, TR10 9EZ UK

**Keywords:** *Acarospora*, Acarosporaceae, Lichen, Metal, Tolerance, Adaptation, Evolution

## Abstract

One of the most successful mechanisms enabling fungi to survive in extreme subaerial environments is by formation of mutualistic symbioses with algae and/or cyanobacteria as lichens. Collections, field and mineral weathering studies and developments in modern instrumental and analytical techniques have considerably advanced knowledge in understanding tolerance mechanisms to stress, environmental adaptation, species concepts and evolutionary processes in lichens colonising metalliferous habitats. This review focuses on the predominantly saxicolous, crustose, taxonomically notoriously challenging *Acarospora* sens. lat. Pioneering studies investigating element and substance localization in *Acarospora* sens. lat. in different geological terrains led to the discovery of novel fixation mechanisms, new minerals and substances associated with lichens, and new taxa and evolutionary lineages. *Acarospora* sens. lat. are generally under-represented in collections. Systematic sampling of *Acarospora* sens. lat. and other saxicolous lichens, in different mineralogical environments is now required, a priority being those occurring in extreme habitats at risk from climatic and other environmental changes. The potential for the discovery of new lichen and mineral species associated with *Acarospora* and other saxicolous crustose lichens, is high. These may represent special mechanisms to tolerate metal toxicity and other forms of environmental stress, including photoprotection.

**Electronic supplementary material:**

The online version of this article (doi:10.1186/1999-3110-55-23) contains supplementary material, which is available to authorized users.

## Review

### Introduction

**Figure 1:**
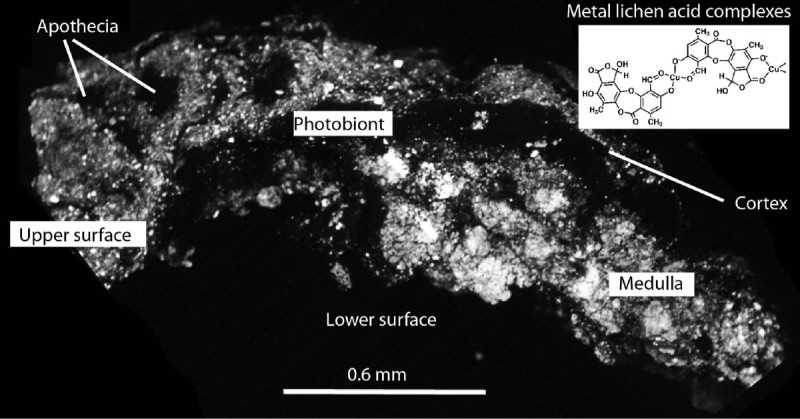
**Back-scattered electron image of** ***Acarospora*** **sampled near Zlatna smelter, Romania (Figure**3**b) showing extensive particulate matter (bright phases) on upper surface and within medulla.** Possible metal uptake mechanisms and metal localization in *Acarospora* include: **(1).** ***Upper surface***. Fixation of particles (mineral and combustion-derived) in dry and wet deposition, metal ion sorption via soluble phases, particulate entrapment in intercellular spaces. **(2).** ***Cortex.*** *ca* 30 μm thick. Particulates are not necessarily inert and may be solubilized by acid precipitation and/or lichen-derived organic acids leading to metal sorption to e.g. extracellular hydrophilic *β*-glucans secreted by mycobiont cortical cells with negatively charged anionic sites [e.g. (Sarret et al., 1998)]; extracellular oxalate and metal lichen acid-complex formation (Cu-Norstictic acid shown) (Purvis et al., 1987; Purvis et al., 1990; Takani et al., 2002); melanins are often present which may sorb metals (U, Cu and Fe) in melanized tissues (McLean et al., 1998; Purvis et al., 2004) **(3).** ***Photobiont Layer***. Intracellular phytochelatin, thiol peptides containing metal-chelating sulphydryl groups of cysteine help protect photobionts from metal toxicity (Pawlik-Skowrońska et al., 2006). **(4)** ***. Medulla***. Particles (mineral, combustion-derived and biogenic oxalates) trapped in intercellular spaces of fungal hyphae and/or attached to medullary hyphae coated with hydrophobic mycobiont-derived secondary metabolites which may further act as sites for metal complexation (Purvis et al., 1987; Purvis et al., 1990). **(5).** ***Lower surface.*** Rhizines occupy by far the bulk of the thallus in section and may extend to several millimetres, hyphae to several centimetres in the substrate. Particles and metals may also be removed from thalli by a variety of processes.

One of the most successful mechanisms enabling fungi to survive in extreme subaerial environments is by formation of mutualistic symbioses with algae and/or cyanobacteria as lichens, where the phototrophs provide a source of carbon and are protected to some degree from light and other external factors (Gadd, 2011). Complex symbiotic associations, bacteria and other microorganisms may also be involved. Primary colonists of rocks, lichens dominate approximately 6% of the Earth’s land surface, and are particularly well represented in the polar regions where they play a major role in the biogeochemical cycling of elements (Figure 1) and contribute to soil formation (Nash, 2008). Metals and their compounds interact with fungi in various ways depending on the metal species, organism, and environment, while fungal metabolism also influences metal speciation and mobility. Even metals essential for life can exert toxicity when present above certain threshold concentrations (Gadd, 2011). In common with all organisms, lichen species have different and often complex ecological requirements, some being restricted to siliceous, calcareous and metal-rich rocks in particular climates (Brodo, 1973; Hawksworth 1973; James et al., 1977). Ecophysiological studies emphasise epiphytic macrolichens often employed in biomonitoring surveys and terricolous lichens typically associated with acid heathland colonizing metal-contaminated sites (Purvis and Pawlik-Skowrońska, 2008). Despite potential toxicity, many lichenized fungi survive and grow in metalliferous habitats, including high montane or polar species, but which also apparently occur in lowland situations where they are restricted to particular mineralogical/geochemical environments (Purvis and Halls, 1996; Bačkor and Bodnárova, 2002; Paukov, 2009,2009; Bielczyk, 2012). Some may be rare and endangered, though in many cases their taxonomic identity is uncertain as monographic treatments and molecular studies have yet to be undertaken. Ecophysiological studies of crustose lichens are usually difficult because of the low biomass and their intimate association with the substrate (e.g. Bačkor and Fahselt, 2004). Energy-dispersive X-ray (EDX) microanalysis is an effective method to study metal fixation in small samples requiring a minimal amount of material (Williamson et al., 1998; Bačkor and Fahselt, 2004). A wide range of complementary microscopical and other surface analytical techniques have successfully been applied to investigate saxicolous lichen biodiversity in different mineralogical terrains, particularly in cold deserts which emphasise the complexity of associations between lichens and other organisms with mineral components and organics (e.g. de los Rios et al., 2004). Alteration of bedrock minerals by lichens and associated organisms and synthesis of biominerals on, within and in the proximity of lichens highlights their significance in mineral nutrient cycling (Banfield et al., 1999; Gadd, 2011).

**Figure 2:**
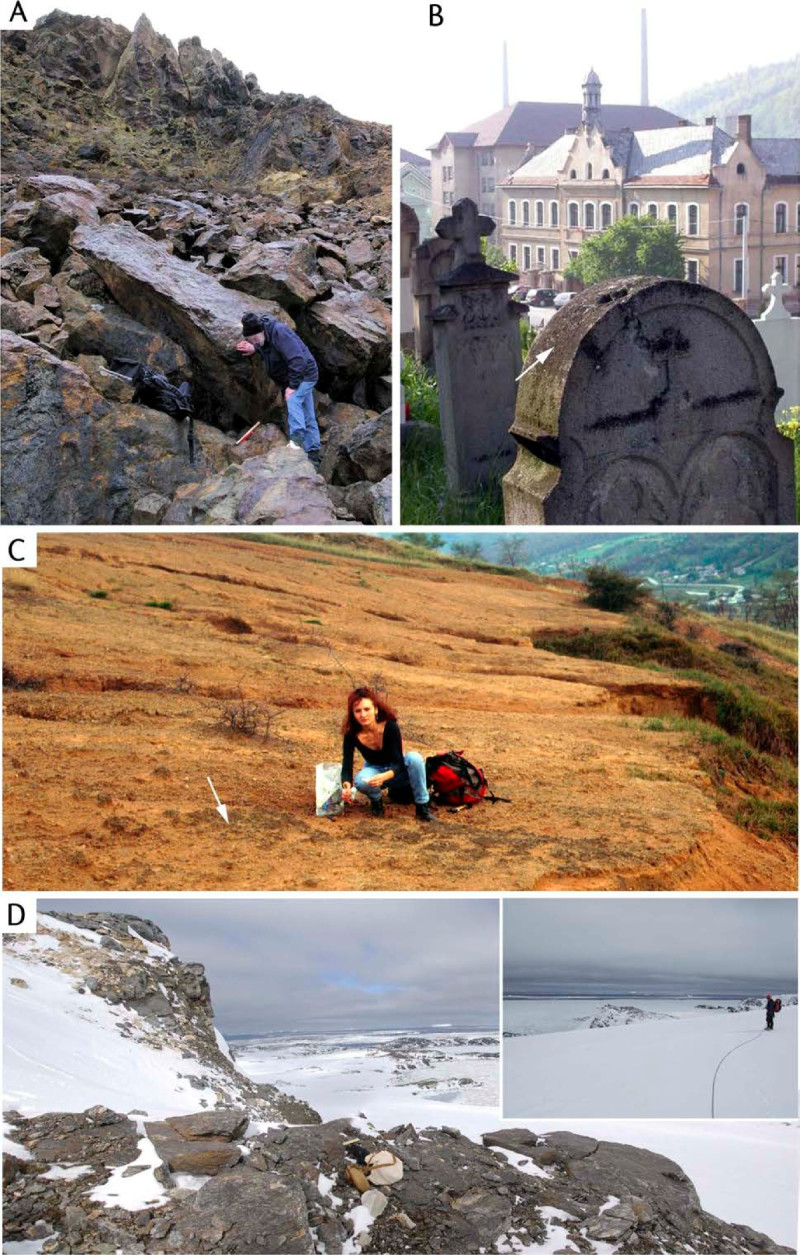
***Acarospora*** **species (arrowed) colonize a range of extreme habitats with differing geology influenced by metals such as (A) acidic, iron sulphide mineralised boulders and mine spoil at Parys Mountain, 24 March 2008; (B) on calcareous, nutrient-enriched tombstones, 12 May 2004 and (C) denuded, metal-enriched soils in a region heavily influenced by acidic and particulate emissions from the former smelter at Zlatna, Romania, 1996 and (D) iron-stained quartz mica schists on the lower slopes of a recently exposed nunatak on the McLeod Glacier, Signy Island, South Orkney Islands.** Inset approaching the nunatak across McLeod Glacier. 16 November 2009.

**Figure 3:**
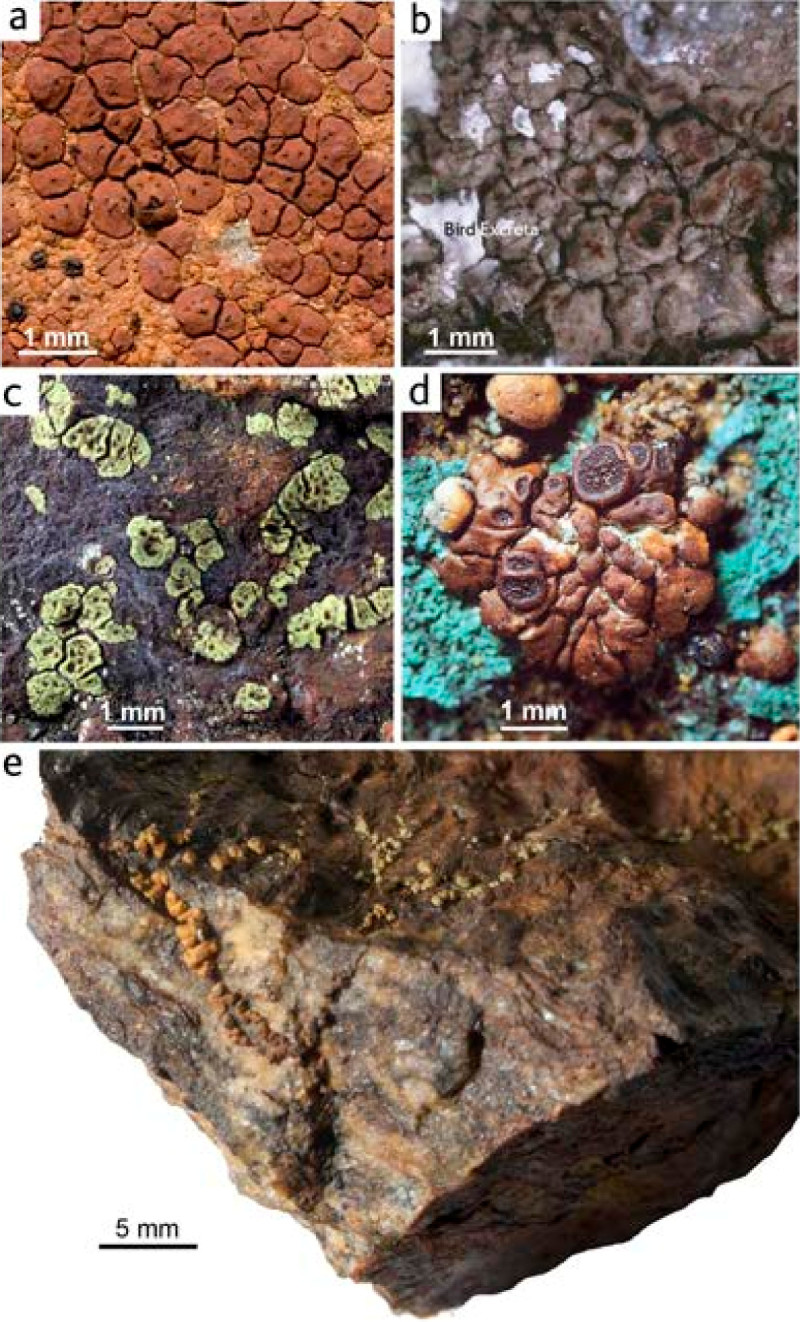
***Acarospora*** **species exhibit a wide range of pigmentation e.g. (A)** *Acarospora sinopica* (deep red-brown) intermixed with *Rhizocarpon oederi* (orange-red), Parys Copper Mountain, (24 March 2008) (hb NHM); **(B)** *Acarospora smaragdula* from concrete fence post adjacent to southern perimeter of unvegetated mine tailings within 500 m of Zlatna smelter, 17 July 1997. **(C)** *Acarospora smaragdula* sampled at Geevor, Cornwall, Purvis and Williamson, 1995 (Spalding et al., 1996) **(D)** dark brown *Acarospora rugulosa* Koerb. colonising brochantite (Cu_4_(SO_4_)(OH)_6_) secondary deposit, greenstone metamorphosed in greenschist to amphibolites facies metamorphism, Ramundberget, Härjedalen, Sweden, O.W. Purvis and R. Santesson, 18 August 1983 (hb: NHM); **(E)** *Acarospora* cf. *badiofusca* on weathered iron-stained schistose rocks, (collection S2_4, O.W. Purvis and B. Maltman), 16 November 2009. Colours ranged from rust-coloured in exposed situations to green in shaded crevices. [Figure 3C adapted from Figure (p. 36) (Spalding et al., 1996); Figure 3D adapted from Figure 1 (Purvis, 1984)].

Pioneer organisms, *Acarospora* taxa colonize rocks, soils, bark, wood and other materials influenced by metals (Figure 2). *Acarospora* sens. lat. and other lichens fix metals and other elements present in wet and dry deposition derived from atmospheric and lithospheric sources (Figure 1). *Acarospora* species sens. lat. range in colour from dull-grey, brown to yellow-green (Clauzade et al., 1981; Clauzade et al., 1982) (Figure 3). Although lichen colour is often due to the presence of lichen products of fungal origin (Huneck and Yoshimura, 1996; Elix and Stocker-Wörgötter, 2008), it has long been suspected that it may be affected by the chemical composition of the substrate itself (Hawksworth, 1973). An obvious effect of mineralization on lichens is the strong rust colour occurring in several species within Acarosporaceae. Thus on certain iron-rich rocks, reddish orange ‘oxydated’ thalli occur, some consistently ‘oxydated’, a characteristic for the species, as in the obligately rust-coloured *Acarospora sinopica* (Figure 3A). Rust-coloured species, such as *A. sinopica*, are often assumed to contain ‘rust’, i.e. hydrated iron oxides, though analysis has rarely been carried out. The taxonomic identity of the rust-coloured *Acarospora sinopica* has been a matter of controversy due to morphological similarities with non-rust coloured *Acarospora smaragdula* belonging to a group of taxonomically notoriously difficult crustose lichens. The existence of coloured taxa in *Acarospora* sens. lat. (Figure 3) and other saxicolous lichen genera has led to considerable taxonomic confusion as to whether taxa merit recognition as distinct species or ecotypes of more ubiquitous species (Hawksworth, 1973; Purvis, 1997).

There has been a long tradition of studying lichens, including *Acarospora* sens. lat. found on metalliferous rocks and slags, especially in Central Europe (Hilitzer, 1923; Schade, 1933; Lange and Ziegler, 1963; Poelt and Ullrich, 1964; Noeske et al., 1970; Wirth, 1972; Hauck et al., 2007). The term ‘chalcophile’ (‘copper-loving’) was first used in a lichenological sense (Poelt and Ullrich, 1964) to describe lichens more-or-less restricted to metalliferous rocks, slags and ores. Volkmar Wirth (Poelt and Ullrich, 1972) was the first to suggest that it was the low pH, rather than iron and other metals, that was responsible for their development. Ferrous sulphides are the principal acid-forming constituents of mine spoils which liberate dilute sulphuric acid as a result of bacterially assisted oxidative weathering and which leads to ‘acid mine drainage’, a major source of metal contamination (Jenkins et al., 2000). The Acarosporion sinopicae community, characterised by the obligate rust-coloured *Acarospora sinopica*, is characteristic of such acidic environments (Wirth, 1972) (Figure 2A). With particular reference to sulphide-rich substrata, metamorphosed in greenschist facies, in central Scandinavia, the author’s multi-disciplinary doctoral studies illustrated the complexity of the interrelationships between Cu and Fe minerals, pH and other ecological factors in determining assemblage composition (Purvis, 1985). A new lichen community was described related to cupriferous habitats at near neutral to slightly basic pH where characteristic *Acarospora* taxa occurred, including *A. rugulosa* Koerber (Figure 3D) and distinctive yellow-green *Acarospora* and lichens belonging to other saxicolous lichen genera (*Aspicilia*, *Bellemerea*, *Lecidea*, *Lecanora*) exhibiting a similar colour. The importance of a mineralogical understanding was highlighted, which determines the availability of elements to saxicolous crustose lichens intimately associated with the substratum through weathering processes which may involve the lichen itself (Purvis, 1985; Purvis and Halls, 1996; Purvis, 2000).

Traditionally, ecological factors have been considered as being extremely important in species delimitation leading, in some cases, to a large number of taxa being proposed in some genera, including *Acarospora* sens. lat. (Hawksworth, 1973). Long recognised as being a true challenge for the taxonomist (Westberg et al., 2011) and often difficult to collect, taxonomists working in herbaria with poor material and without the opportunity to examine the variation of populations in the field have sometimes failed to appreciate the extent to which ecological factors can influence their form (Hawksworth, 1973). Understanding the sources of phenotypic variation in lichens, therefore, is critical to the evaluation of criteria for taxonomic delimitation. Such variation may reflect genetic differences. Phylogenetic analysis employing both ‘traditional’ datasets and molecular techniques is now widely accepted as being the most useful method for analysing natural relationships in lichens (Wedin et al., 1998).

Previous reviews emphasise physiology (Brown, 1976; Brown and Beckett, 1984; Brown, 1987; Brown, 1991; Brown et al., 1994, Richardson, 1995), mineral weathering (Jones et al., 1981; Wilson, 1995; Lee, 2000; Adamo and Violante, 2000), biomonitoring (Branquinho et al., 1999; Garty, 2001; Bargagli and Mikhailova, 2002; Purvis, 2010), ecology, conservation and taxonomy (Hawksworth, 1973; Purvis, 1993,1996;1997), biogeochemistry (Purvis and Halls, 1996, Haas and Purvis, 2006) and cellular responses (Purvis and Pawlik-Skowrońska, 2008). This review concerns tolerance, evolution and adaptation in the predominantly saxicolous crustose lichen genus *Acarospora* sens. lat. in different geological settings with reference to photographic quadrat monitoring carried out to monitor *Acarospora sinopica* over the period 1993–2013 at Parys Copper Mountain, south of Amlwch on Anglesey, North Wales. Crustose lichenized fungi in the Acarosporaceae include splendid examples of organisms tolerating extreme conditions, such as stressful pH conditions (Wirth, 1972), high nutrient concentrations around bird colonies (Clauzade et al., 1981; Øvstedal and Smith, 2001). Others tolerate both natural pollution e.g. growing on the isolated volcanically active, South Sandwich Islands archipelago in the southern South Atlantic (Convey et al. 2000) and near the volcano Mount Vesuvius, Italy (Wedin et al., 2009) as well as anthropogenic pollutant sources (Purvis et al., 2000). *Acarospora anomala* tolerates the fungicide Bordeaux mixture (Poelt and Huneck, 1969) and *Acarospora smaragdula*, wood preservatives (e.g Cu-Cr-As) (Purvis et al., 1985). Metalliferous habitats, such as Parys Copper Mine in Wales with volcanic-associated massive sulphide (‘VMS’) ore deposits is predominantly acidic (sulphide weathering) has provided a classic research site to investigate *adaptation*, *tolerance* and *evolution* in organisms, including lichens (Antonovics et al., 1971; Bradley et al., 1981; Purvis, 2010). Major developments in analytical facilities and techniques have occurred over the past 30 years greatly facilitating the analysis of small amounts of material now enabling a wide range of hypotheses and questions considered by the pioneers to be addressed for the first time.

### Tolerance

Tolerance has been defined as (i) the ability of an organism to endure extreme conditions; (ii) the range of an environmental factor within which an organism or population can survive (Lincoln et al., 1998). A variety of mechanisms contribute to tolerance in fungi, including the production of metal-binding proteins, organic and inorganic precipitation, active transport, and intracellular compartmentalization, while major constituents of cell walls, e.g. chitin and melanin, have significant metal-binding abilities (Gadd, 2011). Metal tolerance has been considered in saxicolous crustose lichens since the pioneering studies carried out in relation to lichen communities occurring on abandoned mine tailings or on rocks with naturally occurring high metal concentrations (Lange and Ziegler, 1963). This has been supported by field and laboratory studies where lichens were analysed for their metal content or exposed to metal solutions, weathering studies, documenting metal deposition around pollution sources (Bargagli and Mikhailova, 2002), such studies most thoroughly reviewed across many groups in Antarctica (Bargagli, 2005).

**Figure 4:**
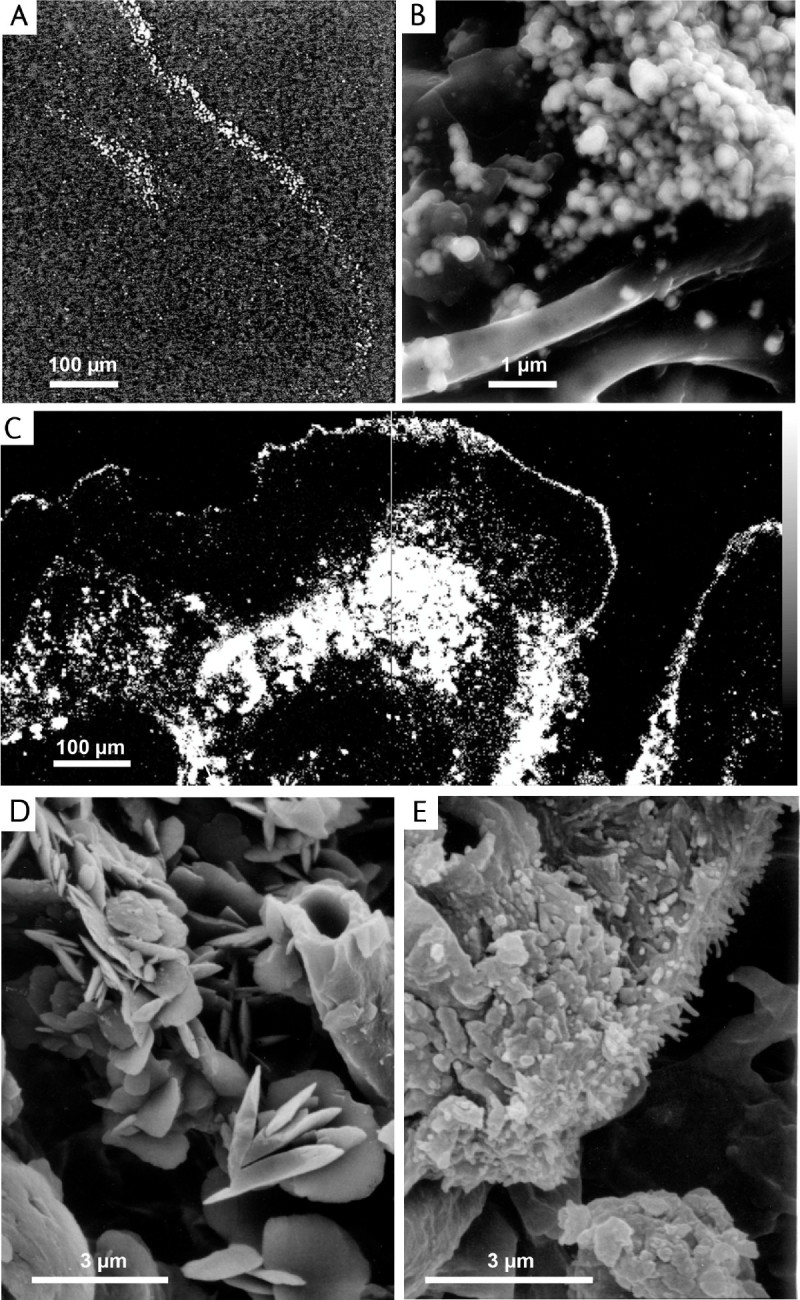
**Developments in SEM played a major role in the discovery of new minerals / mineral phases associated with lichens and potential tolerance mechanisms to environmental stress and in elucidating species concepts. (A)** First x-ray emission map showing Fe localization in the cortex of *Acarospora smaragdula*; **(B-C)** SEM images of *Acarospora smaragdula* agg. sampled from a concrete fencepost subjected to extreme metal particulate fall-out and sulphur dioxide pollution in Zlatna, Romania (see Figure 3B); **(B)** Secondary electron field emission scanning electron microscope (FESEM) image of microbotryoidal granules of Pb/S/Sn phases visible down to 20 nm which may be primary in origin (i.e. of smelter origin) or secondarily mobilized within the lichen. **(C)** X-ray emission map for Pb mainly localized on the surface and medulla. **(D)** SEM micrographs of crystalline inclusions within the medulla of *Acarospora rugulosa* (Figure 2D). **(E)** SEM micrographs of crystals in Cu-rich cortices in *Acarospora smaragdula* (Alstrup 77899). [Figure 4A adapted from Figure 2, Noeske et al., 1970; Figure 4B adapted from Figure 4C, Purvis et al., 2000; Figure 4C adapted from Figure 2A, Purvis et al., 2000; Figure 4d adapted from Figure 2A, Purvis, 1984; Figure 4E adapted from Figure 4B, Purvis et al., 1987.

Sixty years ago Lange and Ziegler investigated the heavy metal content and Fe localization of *Acarospora sinopica* (Wahlenb.) Koerb., *Acarospora smaragdula* v. *lesdainii* f. *subochracea* H. Magn*.* (now *Myriospora dilatata* (M. Westb & Wedin) K. Knudsen & L. Arcadia), *Acarospora montana* H. Magn. and other lichens occurring on slag dumps in the Harz Mountains, Germany. The highest Fe content (55,000 ppm dry weight) was reported in *Acarospora sinopica*, much present as Fe^2+^ in the cortical crust (Figure 4A), possibly present as sulphate, silicate and phosphate (Lange and Ziegler, 1963; Noeske et al., 1970). Lange and Ziegler (1963) proposed that tolerance to metals might involve (i) inherent cytoplasmic tolerance, (ii) cytoplasmic immobilization and detoxification of ions by chemical combination and (iii) transport (or retention) of ions to regions external to the plasmalemma and even the cell wall (Lange and Ziegler, 1963). Since these pioneering studies, further collections, new surveys and application of new techniques emphasise the significance of extracellular immobilzation of ions in *Acarospora* sens. lat.:

Thirty years ago, the author was invited to Härjedalen, Sweden by Rolf Santesson, and examined *Acarospora rugulosa* at an abandoned mine site where it was associated with, and growing on, a range of coloured secondary minerals (  3D), a lichen also found associated with copper mineralization in Nord Trøndelag, Norway (Purvis, 1984; Chisholm et al., 1987). Vivid turquoise inclusions were present within its medulla, visible through cracks on the thallus surface (Figure 3D). In extreme cases almost two thirds of the cross section accounted for the blue phase (Purvis, 1984). The copper content of Swedish material was found to be 16% dry weight, possibly the highest ever recorded. In the SEM, the blue areas consisted of an aggregate of plate-like crystals, 1–5 μm diam. encrusting medullary hyphae, the crystals often so numerous that it was difficult to see the hyphae (Figure 4D). Infra red spectra and XRD from hand-picked material identified the blue phase as being the hydrated copper oxalate, moolooite, CuC_2_O_4_. nH_2_0 (n 0.4-0.7), the first report of this mineral associated with a lichen. The secondary Cu minerals on which the *Acarospora* was growing were identified as the basic chloride, atacamite [Cu_2_Cl(OH)_3_], and the basic Cu sulphate, brochantite [Cu_4_(SO_4_)(OH)_6_] (Figure 3D), both minerals stable under oxidizing alkaline conditions. White included material, consisting of stubby prisms, 1–4 μm long, was identified as being the calcium oxalate, whewellite (CaC_2_O_4_.nH_2_0) (n _~_ 1). However, the appearance of these white crystals was observed to differ from the platy habits of whewellites described in other lichens (Chisholm et al., 1987). It was observed that the lichens containing inclusions did not always grow in direct contact with copper minerals and that reaction of ground or surface water containing Cu with the oxalic acid secreted by the lichen was most likely to lead to the precipitation of copper oxalate hydrate. Studies investigating mineral stability and the interaction between organisms and the lithosphere (e.g. Jones et al., 1981), though not yet focusing on *Acarospora*, have considerably increased knowledge of these complex interactions.

Other oxalates can be expected to occur in *Acarospora* in environments where these have been determined in other lichen genera growing on what have often been considered as ‘unpromising substrates’, including: glushinskite (Mg oxalate dihydrate) in lichen on serpentinite, a rock consisting essentially of magnesian silicate minerals and containing little or no calcium (Wilson et al., 1980), manganese oxalate in lichen on manganese ore (cryptomelane, KMn O_8_O_16_, and powdery lithiophorite Al, Li.MnO_2_OH)_2_ (Wilson and Jones, 1984), ferric oxalate (Ascaso et al., 1982) and lead and zinc oxalates influenced by aerial metal deposition (Sarret et al., 1998). Metal oxalates form part of the potential extracellular metal detoxification mechanisms in lichens and non-lichenised fungi (Purvis, 1984; Wilson and Jones, 1984; Fomina et al., 2006; Purvis and Skowronska, 2008;  l Gadd, 2011). The di-hydrated oxalates of Co, Mg, Fe, Mn, Ni and Zn, completely isomorphous with respect to each other, are likely to occur where lichens colonise suitable substrates thus providing a possible detoxification mechanism to protect the lichen from theses cations. (Wilson and Jones, 1984). Oxalates, where these occur as superficial deposits or as a ‘pruina’ on the thallus surface may help deflect the amount of light reaching lichen photobionts and help lichens survive in extreme environments (Purvis, 2000). However, the taxonomic status of several pruinose taxa requires clarification e.g. British material named as *Acarospora umbilicata* f. *congrediens* with a whitish pruina due to a dense deposit of calcium oxalate found on siliceous walls subjected to calcareous seepage, may represent a pruinose morph of *Acarospora fuscata* (Nyl.) Arnold (Fletcher et al., 2009). Use of the term ‘hyperaccumulator’, as frequently applied to describe the extraordinarily high metal concentrations in the living biomass of vascular plants (van der Ent et al., 2013), is perhaps best avoided in the case of lichens where fixation is extracellular.

A single photobiont species, *Trebouxia jamesii* (Hildreth & Ahmadjian) Gärtner, was determined in different lichen species occurring within a stand of the Acarosporetum sinopicae community sampled at the Schwarze Wand, Salzburger Land, Austria (Beck, 1999). In contrast, up to 3 different *Trebouxia* species were reported in a single thallus in an earlier study of the same community at Parys Mountain, Anglesey, Wales (Douglas et al., 1995). In other stressful habitats, like the Antarctic, there is some suggestion of the presence of a greater range of photobionts in a single lichen species than is found elsewhere (e.g. Romeike et al. 2002). However, further taxonomic study of metal tolerant photobionts is required, related to different chemical types of substrata, as well as their age and degree of ecological succession (Bačkor et al., 2010).

*Acarospora sinopica* and other lichens colonizing Fe-rich habitats have special characteristics in terms of their secondary chemistry. Such species either (i) lack lichen substances, (ii) contain lichen substances with a low affinity to both Fe^2+^ and Fe^3+^ or (iii) contain lichen substances which inhibit Fe^2+^ adsorption, but increase Fe^3+^ adsorption (rhizocarpic acid, norstictic acid). Experimental studies suggest lichen substances play a significant role in Fe adsorption in lichens and determine their tolerance to excess concentrations of Fe (Hauck et al., 2007).

Understanding influences of the chemistry of surface-run off, as so elegantly demonstrated by elegant studies carried out by Hauck and co-workers on epiphytic lichens is also clearly desirable. Cytoplasmic responses, including phytochelatin productions, have not yet been investigated in *Acarospora*. These cysteine-rich peptides derived from glutathione (GSH) are synthesised in response to heavy metals to chelate them intracellularly and important constituents of the complex metal detoxification systems of higher plants, algae and some fungi. They were reported as being produced in an oxidized form by the photobiont in *Lecanora polytropa* (Pawlik-Skowrońska et al., 2006) and would be expected to occur in *Acarospora*.

External localization of a Cu-norstictic acid complex might represent a tolerance mechanism to avoid the toxic effects of Cu in *Acarospora* and other genera (Purvis et al., 1987). This is supported by experimental studies implicating a role for norstictic and other lichen substances in determining tolerance to excess metal ions thus enabling saxicolous lichens to colonise mineralised substrata (Hauck et al., [Bibr CR47][Bibr CR48]). However, other roles, including an adaptive response to illumination cannot be precluded in view of its localization within the cortex above the photobiont layer (see below).

### Adaptation

Adaptation has variously been defined as (i) the process of adjustment of an individual organism to environmental stress; (ii) the process of evolutionary modification which results in improved survival and reproductive efficiency; genotypic adaptation, evolutionary adaptation; (iii) any morphological, physiological, sensory, developmental or behavioural character that enhances the survival and reproductive success of an organism: adaption; adaptive (Lincoln et al., 1998).

Possibly the best example of adaptation in lichens is shown by experimental studies carried out by Yngvar Gauslaa and colleagues on the parietin content of *Xanthoria aureola* growing on coastal rocks in Norway. Lichens at high latitudes experience a substantial annual variation in environmental conditions, including solar radiation. As shown by much work in the Antarctic, lichens form sun-screening pigments produced by the mycobiont to handle high light and UV-B levels (Elix & Stocker-Wörgötter, 2008; Nash, 2008). Such pigments vary seasonally, exhibiting a strong vernal increase and autumnal decline (Gauslaa and McEvoy, 2005). Field observations and analytical studies carried out on *Acarospora* sens. lat. implement both copper lichen complexes and iron compounds as sun-screening pigments.

Several yellow-green norstictic-acid containing saxicolous lichens, belonging to *Acarospora* and other genera were discovered at abandoned mines in Nord Trøndelag, Norway and Ramundberget, Härjedalen, Sweden in the Scandinavian caledonides associated with chalcopyrite (CuFeS_2_) mineralization (Purvis, 1984; Purvis, 1985; Purvis et al., 1985). This unusual coloration had resulted in the description of three yellow *Acarospora* species; *Acarospora isortoquensis* Alstrup, described from Greenland (Alstrup, 1981) and *Acarospora undata* Clauzade, Cl. Roux & V. Wirth from Germany and France (Clauzade et al., 1982) and *Acarospora alberti* Samp. from Portugal (Sampaio, 1920). The association of *Acarospora isortoquensis* with Cu-rich rocks was also noted (Alstrup, 1984). Morphologically similar material was subsequently sampled on Cu-rich epiodiorite on Ben Hope, Sutherland and on fence posts treated with a copper fungicide (Purvis et al., 1985) and at Caradon Hill Copper Mine and Geevor Mine, Cornwall (Spalding, 1996) (Figure 3C). Confirmation of appreciable amounts of copper on the thallus surface and within the upper cortex of the holotypes of *Acarospora isortoquensis*, *Acarospora undata,* the isotype of *A. alberti* and morphologically similar material sampled from Norway, Sweden and UK was obtained by electron probe microanalysis (Purvis, 1984; Purvis, 1985).

**Figure 5:**
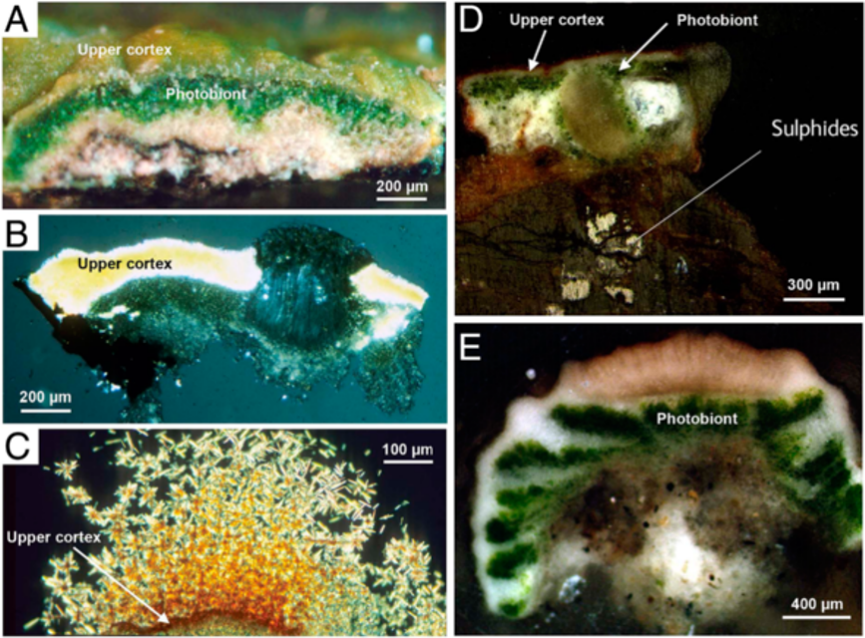
**A-C** ***Acarospora smaragdula*** **(Alstrup 77899). (A)** Section in reflected light; a (black arrow) = pale yellow-green, Cu-rich cortex, b = darker green algal layer; **(B)** squashed section in H_2_O, transmitted light between crossed polars; a = cortex (birefringent), b = medulla (in extinction), c = apothecium (in extinction); **(C)** squashed section, KOH, transmitted light between crossed polars showing red, needle-shaped crystals arising from cortex which has changed red; **(D)** *Acarospora sinopica*, Parys Mountain, resin-embedded section; **(E)** *Acarospora smaragdula* (?*Acarospora amphibola*), Rug Church, on wall beneath metal grille, 19 April 2002. Photobiont arrangement may reflect taxonomy and/or environmental factors. [Figure 5A adapted from Figure 3A, Purvis et al., 1987; Figure 5B adapted from Figure 3B, Purvis et al., 1987.

Early laboratory studies established that soluble metal complexes may form when lichens are allowed to react with suspensions of minerals and rocks (Iskandar and Syers, 1972; Ascaso et al., 1976). Co-localization of copper with nortstictic acid in *A. smaragdula* (Figure 5C) lead to the new hypothesis, that the copper compound could be a Cu-norstictic acid complex (Purvis et al., 1985). Jack Elix synthesised a Cu-norstictic acid complex (Figure 1) and thallus surface scrapings of the holotype of *Acarospora isortoquensis* and similarly coloured lichens colonising cupriferous substrata belonging to other genera were obtained for analysis using XRD and FT-IR. The IR spectra and other studies provided evidence that copper complexing by norstictic acid occurs within the cortex of these lichens and leads to their unusual surface coloration. Structurally related compounds, including psoromic acid, might be expected to form complexes in *Acarospora* where species occur in the appropriate chemical environments, as demonstrated in other genera in Norway (Purvis et al., 1990). Interestingly, lichens containing stictic acid were not observed to adopt this coloration. The aldehyde carbonyl group is present in stictic acid but the adjacent hydroxy group is not, this being *O*-methylated in stictic acid, whereas both are present in norstictic and psoromic acids. This study provided the first evidence for metal complexation with lichen acids in the field and demonstrates the significance of deposition and interaction between cations and co-ordinating ligands in biomass (Haas and Purvis, 2006). The names referring to the yellow-green taxa were usually treated as synonyms of *Acarospora smaragdula* (e.g. Purvis et al., 1985,1987; Santesson et al., 2004).

*Acarospora sinopica* and other lichens colonising iron-rich substrata lack lichen substances which might adsorb iron (Hauck et al., 2007; Westberg et al., 2011). The hypothesis that different compounds were responsible for the different colours observed in samples of deep rust red *Acarospora sinopica and* paler *Acarospora smaragdula ‘f. subochracea’* (now *Myriospora dilatata*) was first investigated in samples from Swedish mines. Modern SEM’s and microanalytical (EPMA, XRD, FTIR) methods were employed to investigate mineralisation in the lichen cortex of the rust red *Acarospora sinopica* and paler yellow-orange *Acarospora smaragdula* f. *subochracea*. Results confirmed the distinctive colours are not simply due to hydrated iron oxides, ‘rust’, as previously believed. Analysis suggests mixed sulphide and oxide phases with little crystallinity, as well as other elements arising from clay minerals are present (Purvis et al., 2008a). A sample of *Acarospora sinopica* from Parys Mountain (Figure 3A) analysed using similar techniques also identified Fe associated with sulphur and oxygen. It highlighted the need for a more detailed study employing a range of mineralogical and micro-analytical techniques, including analytical TEM which will allow mineral characterisation and localisation down to the nanometre scale (Purvis et al., 2008b).

Different iron minerals can be expected to be associated with *Acarospora* and other 'rust’ coloured saxicolous lichens in different environments and according to co-ordinating ligands in biomass. Aluminium-containing goethite was discovered in the rust-coloured *Tremolecia atrata* (Jones et al., 1981). Studies investigating iron plaque formation on plant roots associated with acid mine drainage emphasise geochemical and microbiological processes and tolerance to metal stress (e.g. Batty et al., 2000; Batty, 2003; Kusel et al., 2003). The small inconspicuous lichen, *Acarospora* cf. *badiofusca*, was discovered colonizing iron-stained, quartz mica-schists on the lower slope of Manhaul Rock, a recently exposed nunatak on the McLeod Glacier, Signy Island (Purvis et al., 2013). Its colour ranged from rust on exposed rock surfaces to paler orange and green in shaded crevices in response to illumination (Figure 3E). The upper thallus surface consisted of sub-micron particulate phases containing Fe, Al and O, suggesting mixed oxide/hydroxide phases are present and play a role in photoprotection (Purvis et al., 2013). How iron is transported through the wall of the cortex cells in order to be deposited outside the cell wall, and if oxidation is subsequently an external and ‘inorganic’ process, is unknown (Otto Lange, pers comm.).

**Figure 6:**
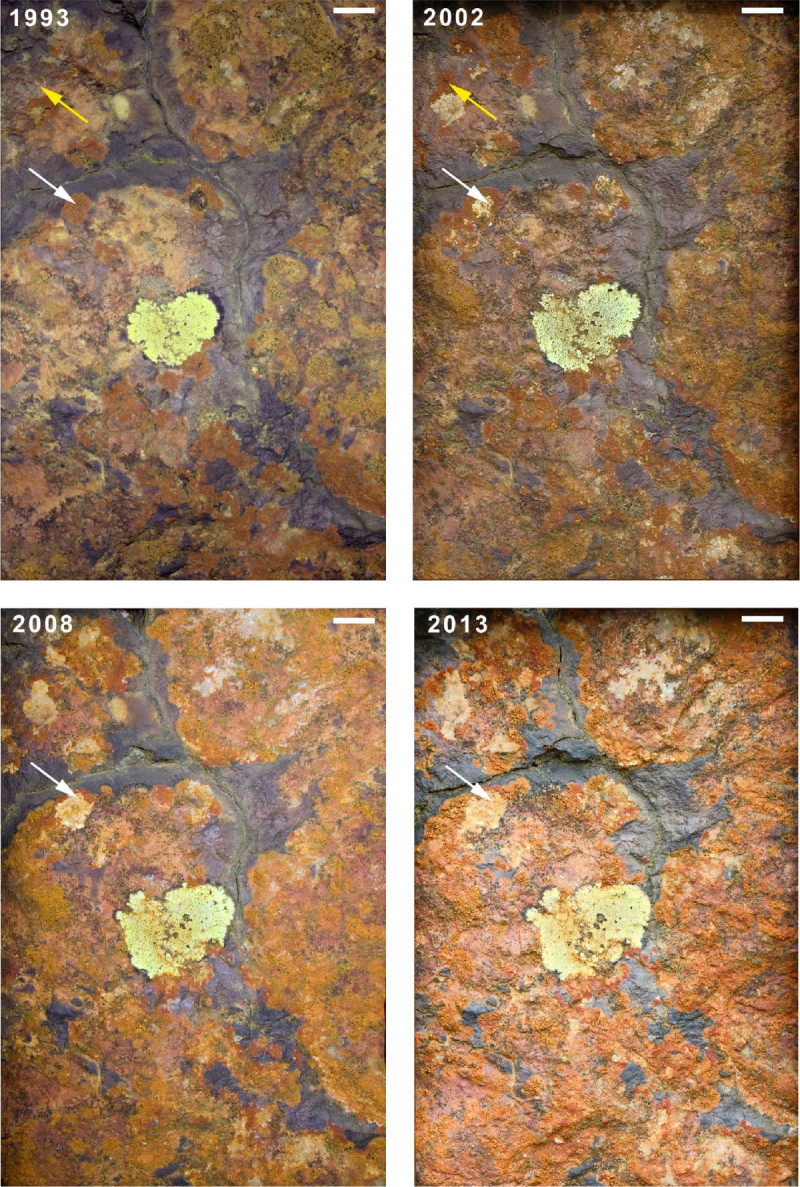
**Photographic monitoring quadrat showing lichen colonization [** ***Rhizocarpon geographicum*** **agg. (yellow-green),** ***Acarospora sinopica*** **(red-brown) and** ***Rhizocarpon oederi*** **(orange-brown)] in 1993, 2002, 2008 and 2013 at Parys Copper Mountain, Anglesey, Wales.** Images manipulated in photoshop to correct for parallax and different camera systems. Yellow arrow indicates ‘thallus expansion’ and white arrow ‘thallus contraction’.

Some lichens, stress tolerators, sensu (Grime, 1979) are notoriously slow growing. Long term photographic monitoring indicates barely perceptible growth over 20 years in alpine situations (Frey, 1959) and over a 25 year period in extreme stressed Antarctic conditions (Sancho et al., 2007). Lichens associated with metalliferous habitats may similarly be extremely slow growing. Mynydd Parys Cu-Pb-Zn mines dominated copper production in the early industrial revolution and nowadays support a specialised microbiology including metallophyte lichens (Jenkins et al., 2000; Purvis, 2010). Eight sites were selected here as sites for designation as Sites of Special Scientific Interest (SSSI) for lichens in 1987 based on the presence of *Acarospora sinopica* and associated species (Purvis, 2010). Photographic quadrat monitoring at Parys Mountain was initiated in 1993 with a focus on *A. sinopica* to assess lichen growth and changes in assemblage composition (Figure 6). Over the 20 year period, *A. sinopica* thalli exhibited both new growth and a contraction of growth with new growth generally being restricted to the outer rims and previously colonised areas towards the thallus centre remaining uncolonised. Co-occurrence with the paler rust-coloured *Rhizocarpon oederi* indicates complex interactions. The similar extent of the dark black mineralised lichen-free zone over the 20-year monitoring period is striking. Dark coloured rocks with low conductivity will have a high propensity for high surface temperatures and may influence lichens and weathering (André et al., 2004).

Physiological adaptation to metals has been especially studied in macrolichens which are more amenable to experimental research both in the field and laboratory, and has not yet been investigated experimentally in *Acarospora* or other saxicolous crustose lichens. The concept of avoidance as an adaptive biological mechanism to allow lichens to resist or tolerate higher metal concentrations of metals than do non-adapted races/ecotypes of species is well established in vascular plants (Baker, 1981). Lack of Fe-adsorbing lichen substances was suggested as a mechanism to tolerate excess concentrations of Fe in *Acarospora sinopica* and other lichens growing on Fe-bearing substrata (Hauck et al., 2007).

### Evolution

The process of evolution has been identified as (i) any directional change; unfolding; (ii) any cumulative change in the characteristics of organisms or populations from generation to generation; descent or development with modification; evolve (Lincoln et al., 1998). For many years the ecological importance and possible evolutionary significance of the chemical components of the upper cortex in lichens has been underestimated (Elix & Stocker-Wörgötter, 2008).

**Figure 7:**
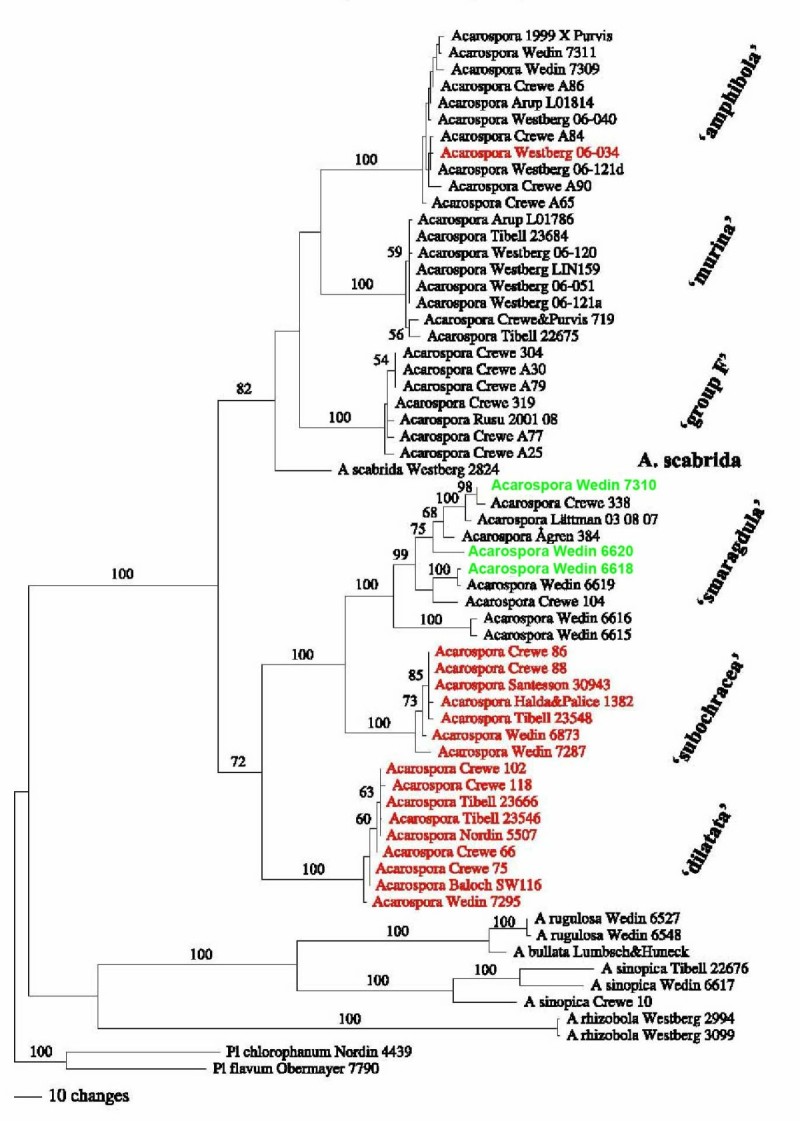
**Phylogeny of the** ***Acarospora smaragdula*** **species complex.** One of four most parsimonious trees resulting from analysis of the combined data matrix, with jackknife values (> 50%) at relevant nodes. The seven species recognized in the species complex are indicated at the right. Iron-fixing samples in red; copper-fixing samples in green [adapted from Figure 1 (Wedin et al., 2009)].

Considerable progress has been made in both clarifying species concepts as well as in understanding evolutionary processes in variously coloured taxa within Acarosporaceae (Crewe et al., 2006; Wedin et al., 2009; Westberg et al., 2011). Due to similarities in areole morphology and abundant punctiform apothecia, the relationship between the obligate rust-coloured *Acarospora sinopica* and typically non-rust coloured, *Acarospora smaragdula*, both described by Wahlenberg (Acharius, 1803) has been in doubt. Many 19th and early 20th century lichenologists treated *Acarospora sinopica* (Purvis, as an infraspecific taxon under *Acarospora smaragdula*^1985^; Crewe et al., 2006). Molecular phylogenetic methods using nuITS-LSU and mtSSU rDNA sequence datasets confirmed that *Acarospora sinopica* as belonging to *Acarospora* s. str. and not to be closely related to *Acarospora smaragdula* (Crewe et al., 2006). Molecular phylogenetic methods were also applied to unravel species concepts and understand evolution in *Acarospora smaragdula* sens. lat (Figure 7). Atypically green, copper-accumulating taxa of *A. smaragdula* sampled from abandoned mines in Sweden, often given species rank, do not form a distinct group but are nested within *A. smaragdula* s. str., indicating that this ability is widespread in this species. In contrast, rust-coloured, iron-accumulating samples form two well supported separate groups, indicating that two morphologically distinct, obligate, iron-accumulating species, are present, but facultatively iron-accumulating populations occur in at least one additional species (Figure 7). The extent to which this reflects phylogeny and/or environmental conditions, however is not known. Micro-organisms may also be involved. The new genus *Silobia* was erected to accommodate the *Acarospora smaragdula* complex and the new species, *Silobia dilatata* (= *Myriospora dilatata* (M. Westb & Wedin) K. Knudsen & L. Arcadia described to accommodate an obligate rust coloured taxon with broadly expanded apothecial discs lacking secondary products. An earlier name *Myriospora* now takes precedence (Arcadia and Knudsen, 2012). Further taxa remain to be described including taxa from Parys Mountain, Wales and Zlatna, Romania.(Figure 7, group F). Rust-red (exposed) and paler orange and green (shaded) samples of *Acarospora* c.f. *badiofusca* (Nyl.) Th. Fr. from Signy Island (Purvis et al., 2013) are being investigated by molecular and microscopical studies to determine if these represent the same species.

## Conclusions

Considerable advances in our knowledge of possible tolerance mechanisms, adaptive responses and evolution in Acarosporaceae and other saxicolous crustose lichens associated with metalliferous habitats (especially in sulphide-rich terrains dominated by iron and copper sulphides) have occurred since the pioneering, inspirational studies carried out investigating element localization in *Acarospora* sens. lat. 60 years ago (Lange and Ziegler, 1963; Noeske et al., 1970). This has been achieved through field studies yielding further collections and advances in modern instrumental and analytical methods and techniques.

Spoil heaps provide refugia for species influenced by former mining and smelting activities (some dating back the bronze age) including some formerly restricted to naturally mineralised rocks but which have been mined away and others, as in the case of vascular plants, which are more characteristic of higher altitudes in montane situations (Purvis, 1993). Targeted sampling of *Acarospora* sens. lat. and associated soils and rocks from other mineralogical and geochemical environments, especially in areas where lichens are generally well developed and at risk from climate change, will yield new collections. New lichen - mineral associations and novel adaptations to tolerate environmental stress will be discovered using established, improved and novel instrumental and analytical techniques. Research should be considered in relation to the biogeochemical significance of fungi. Geochemical transformations that take place and influence plant productivity and the mobility and speciation of toxic elements, are of considerable socioeconomic relevance (Gadd, 2011). New species can be expected to occur in isolated habitats, especially where long-distance dispersal is limiting. As physiological parameters do not necessarily help explain field distributions of lichens in metalliferous habitats (Bačkor et al., 2010), a challenge, at least in terms of understanding potential stressors, is to devise practical field experiments minimising other environmental influences.

## Authors’ original submitted files for images

Below are the links to the authors’ original submitted files for images. Authors’ original file for figure 1 Authors’ original file for figure 2 Authors’ original file for figure 3 Authors’ original file for figure 4 Authors’ original file for figure 5 Authors’ original file for figure 6 Authors’ original file for figure 7

## References

[CR1] Acharius E (1803). Methodus qua omnes detectos lichenes secundum organa carpomorpha ad genera, species et varietates redigere atque observationibus illustrare tentavit Erik Acharius (Methodus Lichenum).

[CR2] Adamo P, Violante P (2000). Weathering of rocks and neogenesis of minerals associated with lichen activity. Appl Clay Sci.

[CR3] Alstrup V (1981). Notes on some lichens and lichenicolous fungi from Greenland. Nord J Bot.

[CR4] Alstrup V (1984). A comparative study of *Acarospora isortoquensis* and *A. undata*. Lichenologist.

[CR5] André M-F, Hall K, Comte V (2004). Optical rock properties and weathering processes in polar environments (with special reference to Antarctica). Polar Geog.

[CR6] Antonovics J, Bradshaw AD, Turner RG (1971). Heavy metal tolerance in plants. Adv Ecol Res.

[CR7] Arcadia L, Knudsen K (2012). The name *Myriospora* is available for the *Acarospora smaragdula* group. Opuscula Philolichenum.

[CR8] Ascaso C, Galvan J, Ortega C (1976). Studies on the pedogenetic action of lichen acids. Pedobiologia.

[CR9] Ascaso C, Galvan J, Ortega C (1982). The weathering of calcareous rocks by lichens. Pedobiologia.

[CR10] Bačkor M, Bodnárova M (2002). Additions to the lichen flora of Slovak Repoublic 1. Thaiszia J Bot Kosiče.

[CR11] Bačkor M, Fahselt D (2004). Using EDX-microanalysis and X-ray mapping to demonstrate metal uptake by lichens. Biologia, Bratislava.

[CR12] Bačkor M, Peksa O, Skaloud P, Baçkorova M (2010). Photobiont diversity in lichens from metal-rich substrata based on ITS rDNA sequences. Ecotox Environ Safe.

[CR13] Baker AJM (1981). Accumulators and excluders - strategies in the response of plants to heavy-Metals. J Plant Nutr.

[CR14] Banfield JP, Barker WW, Welch SA, Taunton A (1999). Biological impact on mineral dissolution: application of the lichen model to understanding mineral weathering in the rhizosphere. Proc Natl Acad Sci USA.

[CR15] Bargagli R (2005). Antarctic Ecosystems: Environmental Contamination, Climate Change, and Human Impact.

[CR16] Bargagli R, Mikhailova I, Nimis PL, Scheidegger C, Wolseley PA (2002). Accumulation of inorganic contaminants. Monitoring with Lichens - Monitoring Lichens, vol 7.

[CR17] Batty LC (2003). Wetland plants: more than just a pretty face?. Land Contamination and Reclamation.

[CR18] Batty LC, Baker AJM, Wheeler BD, Curtis CD (2000). The effect of pH and plaque on the uptake of Cu and Mn in *Phragmites australis* (Cav.) Trin ex. Steudel. Ann Bot.

[CR19] Beck A (1999). Photobiont inventory of a lichen community growing on heavy- metal-rich rock. Lichenologist.

[CR20] Bielczyk U, Lipnicki L (2012). Lichens of zinc-lead post-mining areas in the Olkusz region – state of preservation, threats and needs for protection. Lichen protection – Protected lichen species: 119–128.

[CR21] Bielczyk U, Jędrzejczyk-Korycińska M, Kiszka J (2009). Lichens of abandoned zinc-lead mines. Acta Mycologica.

[CR22] Bradley R, Burt AJ, Read DJ (1981). Mycorrhizal infection and resistance to heavy-metal toxicity in *Calluna vulgaris*. Nature.

[CR23] Branquinho C, Catarino F, Brown DH, Pereira MJ, Soares A (1999). Improving the use of lichens as biomonitors of atmospheric metal pollution. Sci Total Environ.

[CR24] Brodo IM, Ahmadjian V (1973). Substrate Ecology. The Lichens.

[CR25] Brown DH, Brown DH, Hawksworth DL, Bailey RH (1976). 17. Mineral uptake by lichens. Lichenology: Progress and Problems.

[CR26] Brown DH, Peveling E (1987). The location of mineral elements in lichens: implications for metabolism. Progress and Problems in Lichenology in the Eighties.

[CR27] Brown DH (1991). Lichen mineral studies - currently clarified or confused. Symbiosis.

[CR28] Brown DH, Beckett RP (1984). Uptake and effect of cations on lichen metabolism. Lichenologist.

[CR29] Brown DH, Avalos A, Miller JE, Bargagli R (1994). Interactions of lichens with their mineral environment. Cryptogam Bot.

[CR30] Chisholm JE, Jones GC, Purvis OW (1987). Hydrated copper oxalate, moolooite, in lichens. Mineral Mag.

[CR31] Clauzade G, Roux C, Rieux R (1981). Les Acarospora de l’Europe occidentale et de la région méditerranéenne. Bull Mus Hist Nat Marseille.

[CR32] Clauzade G, Roux C, Wirth V (1982). *Acarospora undata* sp. nov. Bull. Mus. Hist. Nat. Marseille.

[CR33] Convey P, Lewis Smith RI, Hodgson DA, Peat HJ (2000). The flora of the South Sandwich Islands, with particular reference to the influence of geothermal heating. J Biogeogr.

[CR34] Crewe AT, Purvis OW, Wedin M (2006). Molecular phylogeny of Acarosporaceae (Ascomycota) with focus on the proposed genus *Polysporinopsis*. Mycol Res.

[CR35] De los Rios A, Wierzchos J, Sancho LG, Ascaso C (2004). Exploring the physiological state of continental Antarctic endolithic microorganisms by microscopy. FEMS Microbiol Ecol.

[CR36] Douglas GE, John DM, Purvis OW (1995). The identity of phycobionts from lichens in the Acarosporion sinopicae alliance. The Phycologist.

[CR37] Elix JA, Stocker-Wörgötter E (2008). Biochemistry and secondary metabolites.

[CR38] Fletcher A, James PW, Purvis OW, Smith CW, Aptroot A, Coppins BJ, Fletcher A, Gilbert OL, James PW, Wolseley PA (2009). The Lichens of Great Britain and Ireland.

[CR39] Fomina MA, Burford EP, Gadd GM, Gadd GM (2006). 10. Fungal dissolution and transformation of minerals: significance for nutrient and metal mobility. Fungi in biogeochemical cycles.

[CR40] Frey E (1959). Die Flechtenflora und -vegetation des Nationalparks in Unterengadin: II: Die Entwicklung der Flechenvegetation auf photogrammetrisch kontrollierten Dauerflächen; Ergebn. wiss. Unters Schweiz Nat Parks.

[CR41] Gadd GM, Reitner J, Thiel V (2011). Geomycology. Encyclopedia of Geobiology, Part 7.

[CR42] Garty J (2001). Biomonitoring atmospheric heavy metals with lichens: Theory and application. Crit Rev Plant Sci.

[CR43] Gauslaa Y, McEvoy M (2005). Seasonal changes in solar radiation drive acclimation of the sun-screening compound parietin in the lichen *Xanthoria parietina*. Basic Appl Ecol.

[CR44] Grime JP (1979). Plant Strategies and Vegetation Processes.

[CR45] Haas JR, Purvis OW, Gadd GM (2006). Chapter 15. Lichen Biogeochemistry. Fungi in Biogeochemical Cycles.

[CR46] Hauck M, Huneck S, Elix JA, Paul A (2007). Does secondary chemistry enable lichens to grow on iron-rich substrates?. Flora.

[CR47] Hauck M, Jürgens S-R, Leuschner C (2009). Norstictic acid: correlations between its physico-chemical characteristics and ecological preferences of lichens producing this depsidone. Environ Exp Bot.

[CR48] Hauck M, Jürgens S-R, Willenbruch K, Huneck S, Leuschner C (2009). Dissociation and metal-binding characteristics of yellow lichen substances suggest a relationship with site preferences of lichens. Annals of Botany.

[CR49] Hawksworth DL, Heywood VH (1973). Ecological factors and species delimitation in the lichens. Taxonomy and Ecology. Systematics Association Special Volume No. 5.

[CR50] Hilitzer A (1923). Les lichens des rochers amphiboliques aux environs de Vseruby. Čas Nár muz.

[CR51] Huneck S, Yoshimura I (1996). Identification of Lichen Substances.

[CR52] Iskandar IK, Syers JK (1972). Metal-complex formation by lichen compounds. J Soil Sci.

[CR53] James PW, Hawksworth DL, Rose F, Seaward MRD (1977). Lichen communities in the British Isles: a preliminary conspectus. Lichen Ecology.

[CR54] Jenkins DA, Johnson AH, Freeman C, Cotter-Howells JD, Campbell LS, Valsami-Jones E, Batchelder M (2000). Mynydd Parys Cu-Pb-Zn Mines: mineralogy, microbiology and acid mine drainage. Environmental Mineralogy: Microbial Interactions, Anthropogenic Influences, Contaminated Land and Waste Management.

[CR55] Jones D, Wilson MJ, McHardy WJ (1981). Lichen weathering of rock-forming minerals - application of scanning electron-microscopy and micro-probe analysis. J Microsc-Oxford.

[CR56] Kusel K, Chabbi A, Trinkwalter T (2003). Microbial processes associated with roots of bulbous rush coated with iron plaques. Microb Ecol.

[CR57] Lange OL, Ziegler H (1963). Der schwermetallgehalt von flechten aus dem Acarosporetum sinopicae auf erzschlackenhalden des Harzes. I Eisen und kupfer Mitt Florist-soziol Arb.gem.

[CR58] Lee M, Cotter-Howells JD, Campbell LS, Valsami-Jones E, Batchelder M (2000). Weathering of rocks by Lichens. Environmental Mineralogy: Anthropogenic Influences, Contaminated Land and Waste Management.

[CR59] Lincoln R, Boxshall G, Clark P (1998). A Dictionary of Ecology, Evolution and Systematics.

[CR60] McLean J, Purvis OW, Williamson BJ, Bailey EH (1998). Role for lichen melanins in uranium remediation. Nature.

[CR61] Nash TH (2008). Lichen Biology.

[CR62] Noeske O, Läuchli A, Lange OL, V.G H, Ziegler H (1970). Konzentration und lokalisierung von schwermetallen in flechten der erzschlackenhalden des Harzes. Deut Bot Gesell NF.

[CR63] Øvstedal DO, Smith RIL (2001). Lichens of Antarctica and South Georgia.

[CR64] Paukov AG (2009). The lichen flora of serpentine outcrops in the Middle Urals of Russia. Northeastern Naturalist.

[CR65] Pawlik-Skowrońska B, Purvis OW, Pirszel J, Skowroński T (2006). Cellular mechanisms of Cu-tolerance in the epilithic lichen *Lecanora polytropa* growing at a copper mine. Lichenologist.

[CR66] Poelt J, Huneck S (1969). *Lecanora vinetorum* nova spec., ihre Vergesellschaftung, ihre Ökologie und ihre Chemie. Österr Bot Z.

[CR67] Poelt J, Ullrich H (1964). Über einige chalkophile *Lecanora* - Arten der Mitteleuropäischen Flora (Lichenes, Lecanoraceae). Österr Bot Z.

[CR68] Purvis OW (1984). The occurrence of copper oxalate in lichens growing on copper sulphide-bearing rocks in Scandinavia. Lichenologist.

[CR69] Purvis OW (1985). The Effect of Mineralization on Lichen Communities with Special Reference to Cupriferous Substrata.

[CR70] Purvis OW (1993). The botanical interest of mine spoil heaps - the lichen story. J Russell Soc.

[CR71] Purvis OW (1996). Interactions of lichens with metals. Science Progress.

[CR72] Purvis OW, Claridge MF, Dawah HA, Wilson MR, vol 54 (1997). The species concept in lichens. Species.

[CR73] Purvis OW (2000). Lichens.

[CR74] Purvis OW, Batty LC, Hallberg K (2010). Chapter 3. Lichens and Industrial Pollution. Ecology of Industrial Pollution.

[CR75] Purvis OW, Halls C (1996). A review of lichens in metal-enriched environments. Lichenologist.

[CR76] Purvis OW, Pawlik-Skowrońska B, Avery SV, Stratford M, van West P (2008). Chapter 12. Lichens and Metals. Stress in Yeasts and Filamentous Fungi.

[CR77] Purvis OW, Gilbert OL, James PW (1985). The influence of copper mineralization on *Acarospora smaragdula*. Lichenologist.

[CR78] Purvis OW, Elix JA, Broomhead JA, Jones GC (1987). The occurrence of copper norstictic acid in lichens from cupriferous substrata. Lichenologist.

[CR79] Purvis OW, Elix JA, Gaul KL (1990). The occurrence of copper-psoromic acid in lichens from cupriferous substrata. Lichenologist.

[CR80] Purvis OW, Williamson BJ, Bartok K, Zoltani N (2000). Bioaccumulation of lead by the lichen *Acarospora smaragdula* from smelter emissions. New Phytol.

[CR81] Purvis OW, Bailey EH, McLean J, Kasama T, Williamson BJ (2004). Uranium biosorption by the lichen *Trapelia involuta* at a uranium mine. Geomicrobiol J.

[CR82] Purvis OW, Kearsley A, Cressey G, Crewe AT, Wedin M (2008). Mineralization in rust-coloured *Acarospora*. Geomicrobiol J.

[CR83] Purvis OW, Kearsley A, Cressey G, Batty LC, Jenkins DA, Crewe AT, Wedin M, Chen Z-S, Lee D-Y, Lin T-S (2008). Mineralization in rust-coloured *Acarospora*. Proceedings of the 14th International Conference on Heavy Metals in the Environment.

[CR84] Purvis OW, Convey P, Flowerdew MJ, Peat HJ, Najorka J, Kearsley A (2013). Iron localization in *Acarospora* colonizing schist following glacial retreat on Signy Island. Antarc Sci.

[CR85] Richardson DHS (1995). Metal uptake in lichens. Symbiosis.

[CR86] Romeike J, Friedl T, Helms G, Ott S (2002). Genetic diversity of algal and fungal partners in four species of *Umbilicaria* (Lichenized ascomycetes) along a transect of the Antarctic peninsula. Mol Biol Evol.

[CR87] Sampaio G (1920). Líquenes Inéditos.

[CR88] Sancho LG, Green ATG, Pintado A (2007). Slowest to fastest: extreme range in lichen growth rates supports their use as an indicator of climate change in Antarctica. Flora.

[CR89] Santesson R, Moberg R, Nordin A, Tønsberg T, Vitikainen O (2004). Lichen-Forming and lichenicolous Fungi of Fennoscandia.

[CR90] Sarret G, Manceau A, Cuny D, Van Haluwyn C, Deruelle S, Hazemann JL, Soldo Y, Eybert-Berard L, Menthonnex JJ (1998). Mechanisms of lichen resistance to metallic pollution. Environ Sci Technol.

[CR91] Schade A (1933). Das Acarosporetum sinopicae als Charaktermerkmal der Flechtenflora sächsischer Bergwerkshalden. Sitz Abh Naturwiss Ges Isis Dresden.

[CR92] Spalding A, Edwards T, Sinkins B, Purvis OW, Stewart J (1996). The nature conservation value of metalliferous mining sites.

[CR93] Takani M, Yajima T, Masuda H, Yamauchi O (2002). Spectroscopic and structural characterization of copper(II) and palladium(II) complexes of a lichen substance usnic acid and its derivatives: Possible forms of environmental metals retained in lichens. J Inorg Biochem.

[CR94] van der Ent A, Baker AJM, Reeves RD, Pollard AJ, Schat H (2013). Hyperaccumulators of metal and metalloid trace elements: facts and fiction. Plant Soil.

[CR95] Wedin M, Tønsberg T, Brown DH (1998). ‘Taxonomy, evolution and classification of lichens and related fungi’ 10–11 January 1998. The Lichenologist.

[CR96] Wedin M, Westberg M, Crewe AT, Tehler A, Purvis OW (2009). Species delimitation and evolution of metal bioaccumulation in the lichenized *Acarospora smaragdula* (Ascomycota, Fungi) complex. Cladistics.

[CR97] Westberg M, Crewe AT, Purvis OW, Wedin M (2011). *Silobia*, a new genus for the *Acarospora smaragdula* complex (Ascomycota, Acarosporales) and a revision of the group in Sweden. Lichenologist.

[CR98] Williamson BJ, McLean J, Purvis OW (1998). Application of X-ray element mapping across the lichen-rock interface. J Microsc.

[CR99] Wilson MJ (1995). Interactions between lichens and rocks: a review. Cryptog Botany.

[CR100] Wilson MJ, Jones D (1984). The occurrence and significance of manganese oxalate in *Pertusaria corallina* (Lichenes). Pedobiologia.

[CR101] Wilson MJ, Jones D, Russell JD (1980). Glushinskite, a naturally occurring magnesium oxalate. Mineral Mag.

[CR102] Wirth V (1972). Die Silikatflechten: Gemeinschaften im außeralpinen Zentraleuropa. Dissertationes Botanicae Lehre.

