# Supplementary material for: Adaptation and interaction of saxicolous crustose lichens with metals
Source: Bot Stud. 2014 Feb 4;55:23. doi: 10.1186/1999-3110-55-23 (PMC5430356; doi:10.1186/1999-3110-55-23)
Supplement: Supplementary file 1 — Authors’ original file for figure 1 [file 40529_2013_71_MOESM1_ESM.pdf]

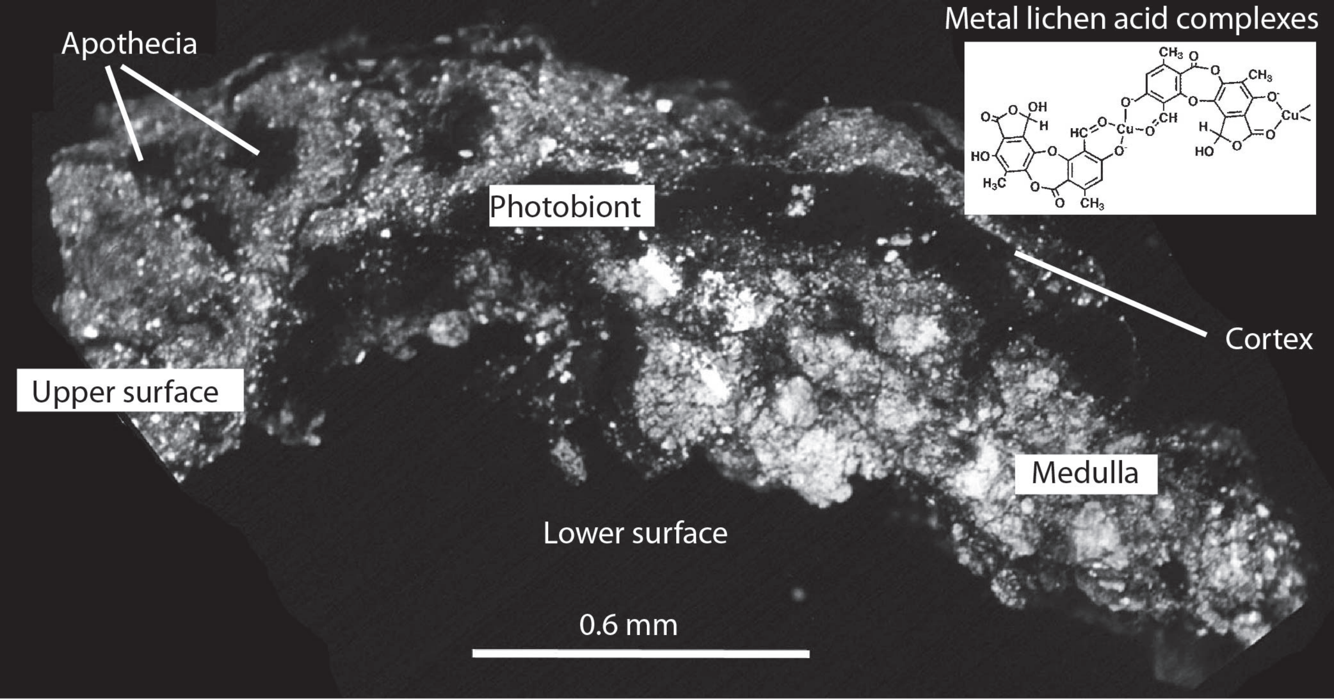
Metal lichen acid complexes
Apothecia
Photobiont
Cortex
Upper surface
Medulla
Lower surface
0.6 mm
