# Supplementary figures and images for: Adaptation and interaction of saxicolous crustose lichens with metals
Source: Bot Stud. 2014 Feb 4;55:23. doi: 10.1186/1999-3110-55-23 (PMC5430356; doi:10.1186/1999-3110-55-23)

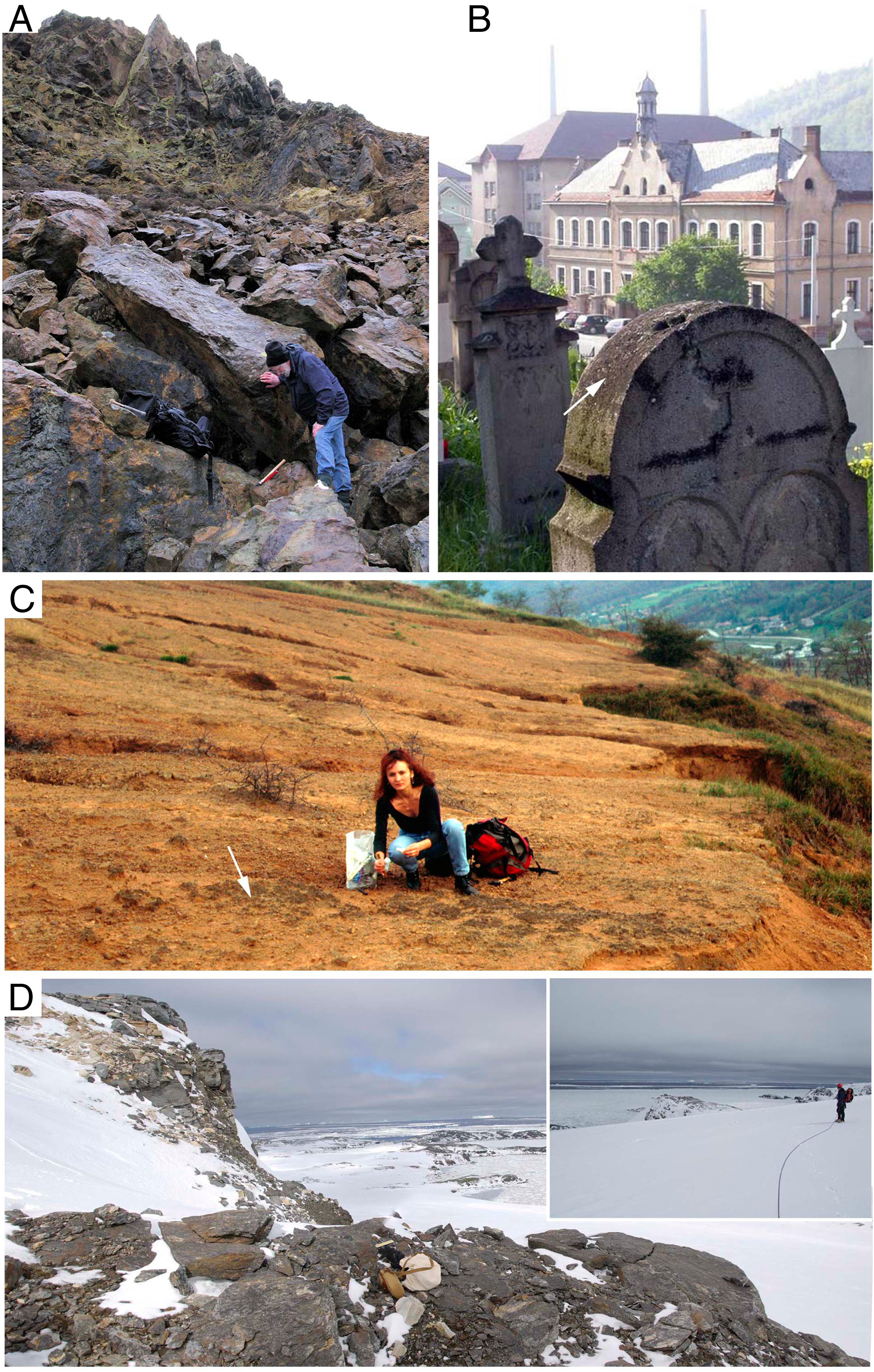

Supplement: Supplementary file 2 — Authors’ original file for figure 2 [file 40529_2013_71_MOESM2_ESM.tiff]

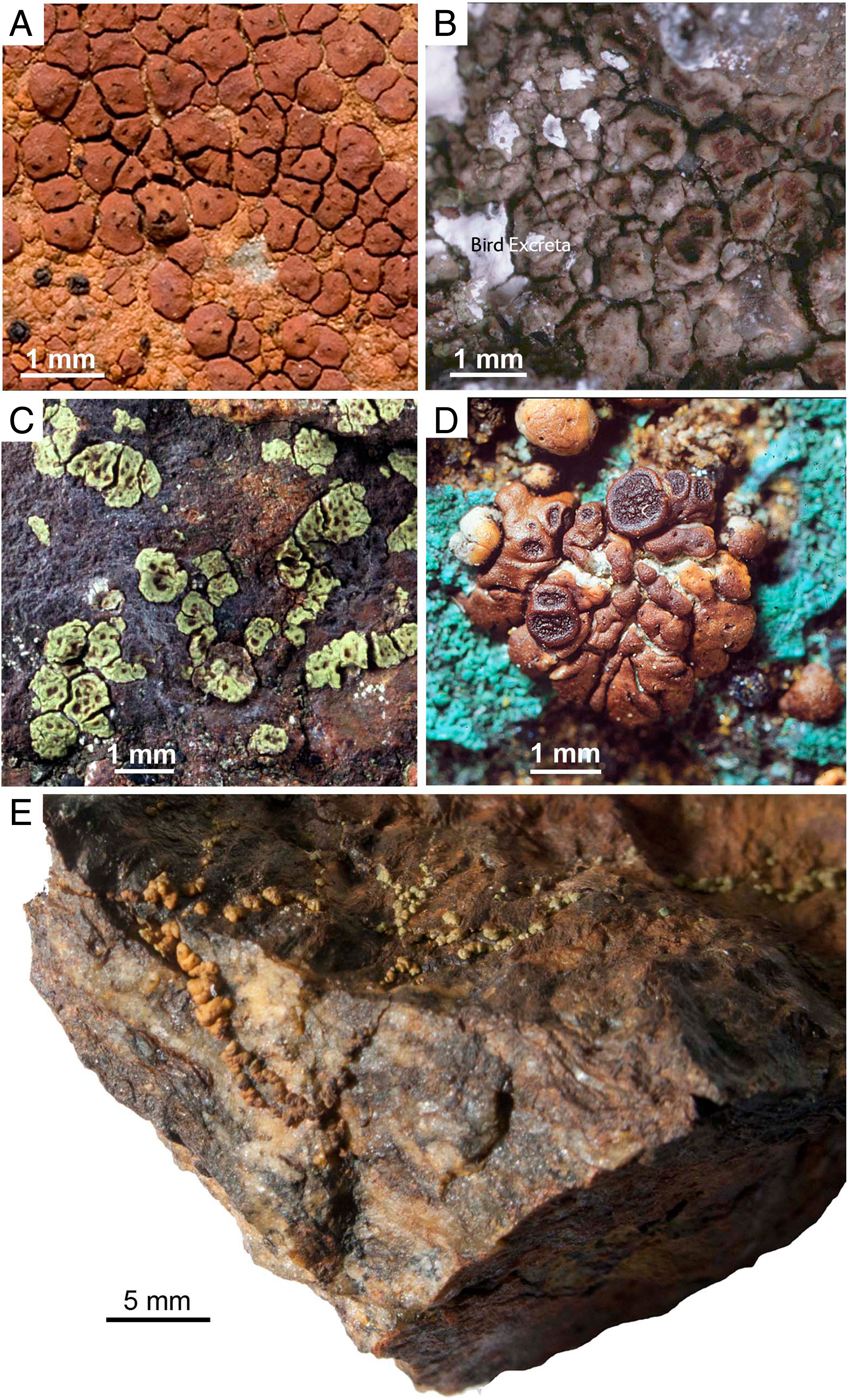

Supplement: Supplementary file 3 — Authors’ original file for figure 3 [file 40529_2013_71_MOESM3_ESM.tiff]

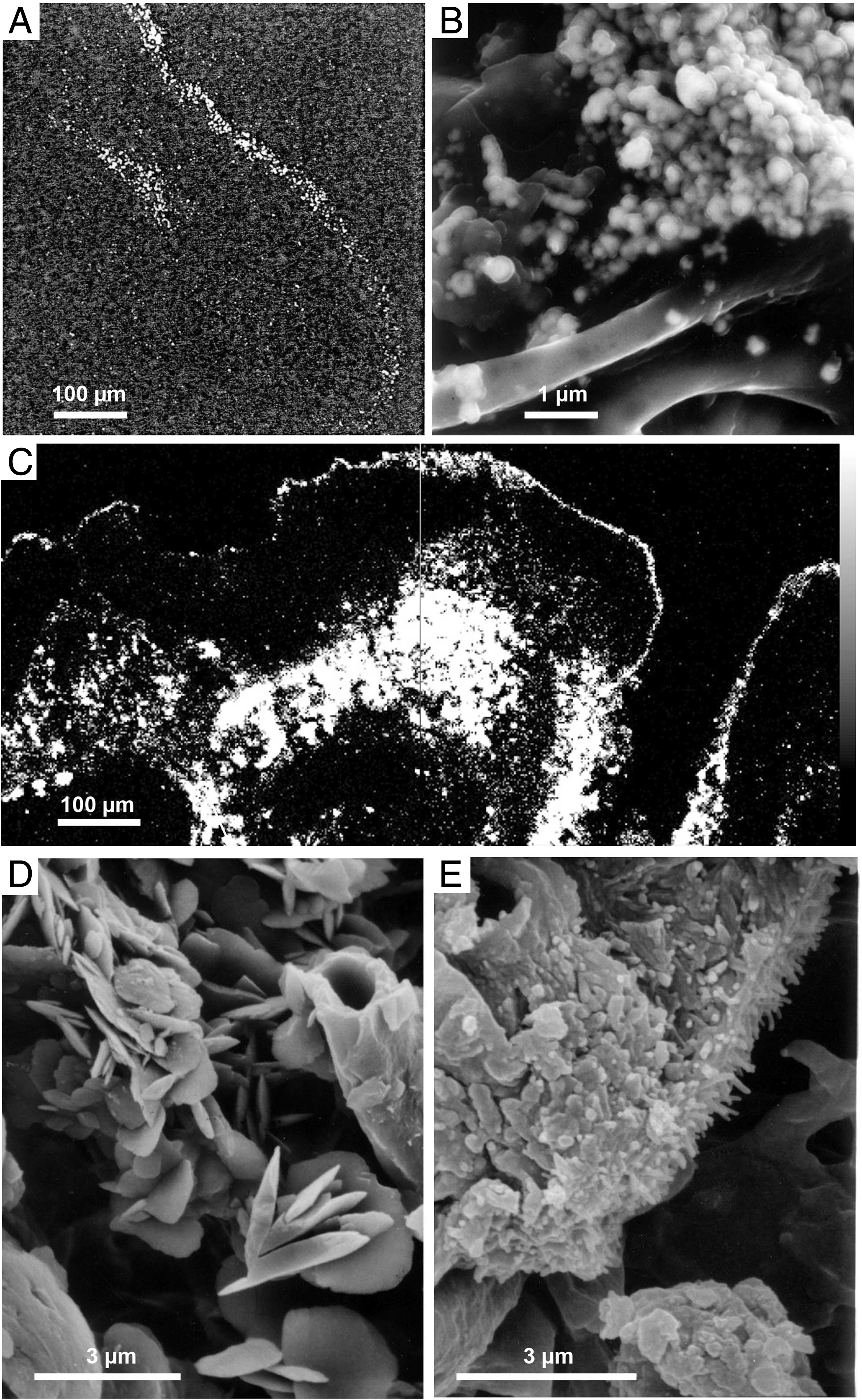

Supplement: Supplementary file 4 — Authors’ original file for figure 4 [file 40529_2013_71_MOESM4_ESM.tiff]

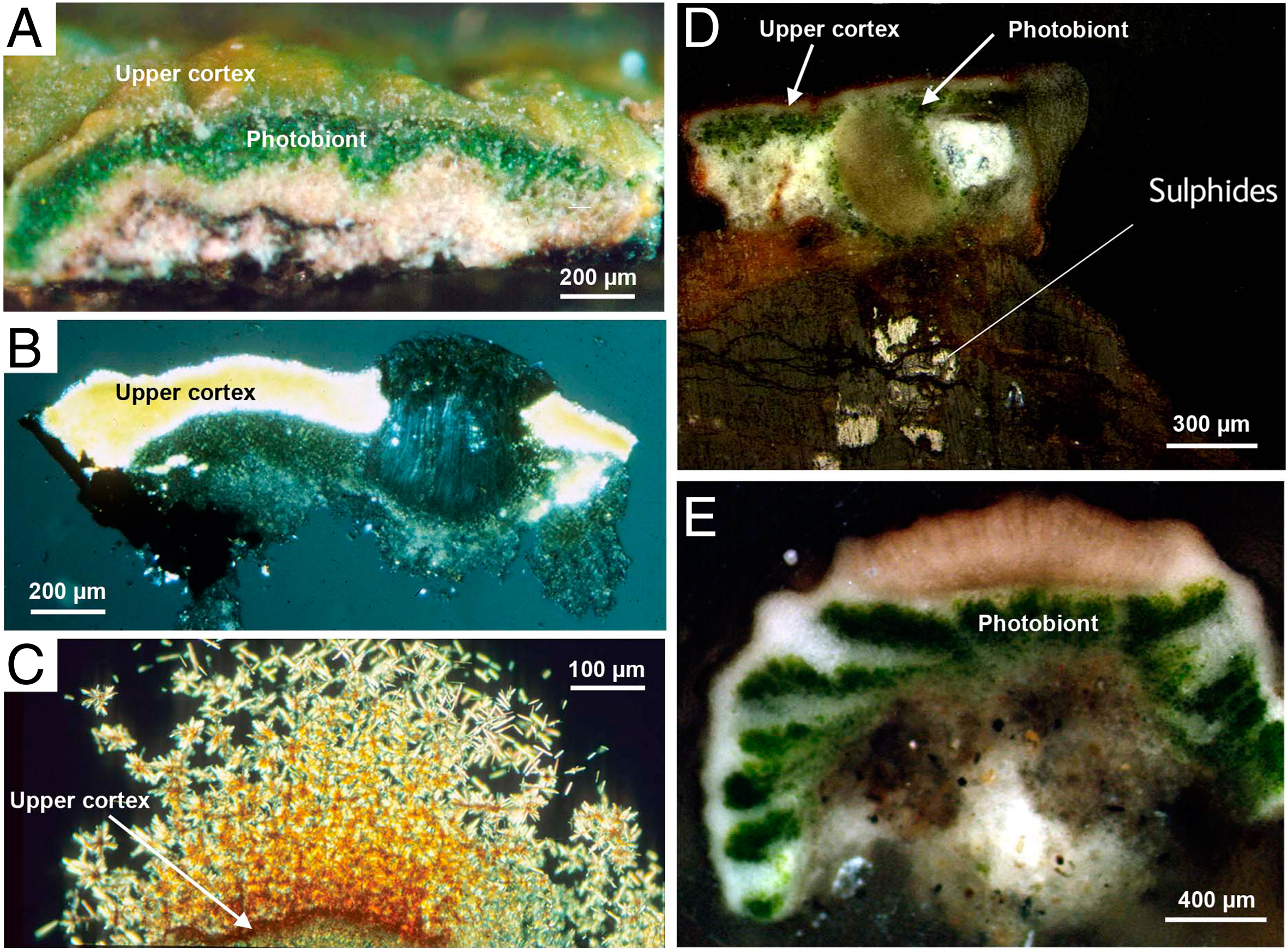

Supplement: Supplementary file 5 — Authors’ original file for figure 5 [file 40529_2013_71_MOESM5_ESM.tiff]

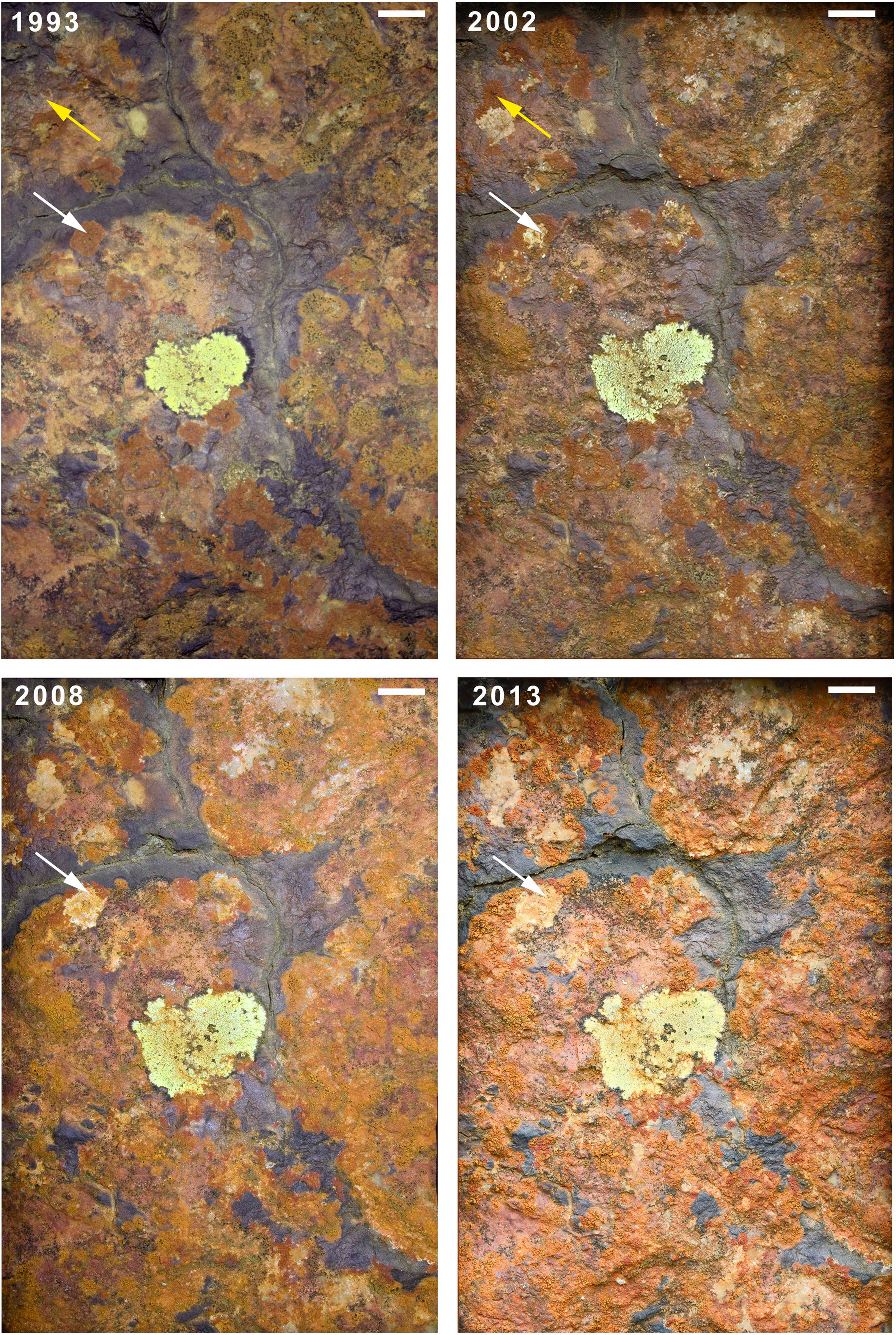

Supplement: Supplementary file 6 — Authors’ original file for figure 6 [file 40529_2013_71_MOESM6_ESM.tiff]

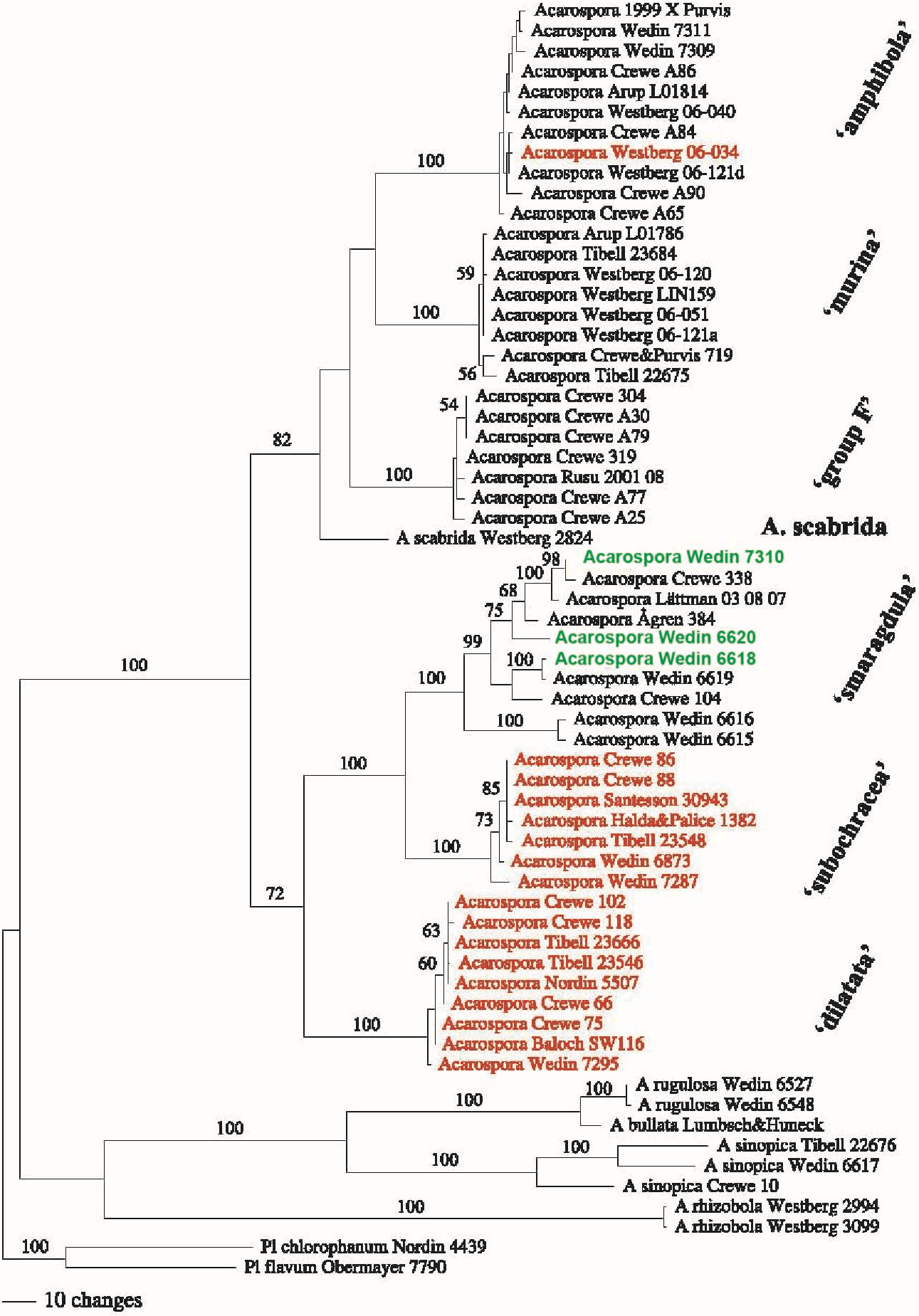

Supplement: Supplementary file 7 — Authors’ original file for figure 7 [file 40529_2013_71_MOESM7_ESM.tif]
